# The Fellowship of the Royal College of Radiologists (FRCR) examination: a review of the evidence

**DOI:** 10.1016/j.crad.2018.09.005

**Published:** 2018-12

**Authors:** T.C. Booth, R.deP.M. Martins, L. McKnight, K. Courtney, R. Malliwal

**Affiliations:** a Department of Neuroradiology, King's College Hospital NHS Foundation Trust, London, SE5 9RS, UK; b School of Biomedical Engineering and Imaging, King’s College London, Rayne Institute, 4th Floor, Lambeth Wing, St. Thomas' Hospital, SE1 7EH, London; c Department of Radiology, Queen Alexandra Hospital, Portsmouth PO6 3LY, UK; d Department of Radiology, Morrison Hospital, Swansea SA6 6NL, UK; e Department of Radiology, King's College Hospital NHS Foundation Trust, London SE5 9RS, UK

## Abstract

The Fellowship of the Royal College of Radiologists (FRCR) examination is the professional qualification that is essential for career progression in clinical radiology within the UK. It is also important for career progression in many countries internationally. The FRCR has evolved and changed over the last decade. In this systematic review we appraise and summarise the available data relating to the FRCR emphasising the published evidence regarding the validity, reliability, and acceptability of this examination. Comparison is made to other equivalent medical examinations, as well as a more recently published commissioned external review of the FRCR examinations. The Clinical Radiology Part 2B (CR2B) examination in its pre-existing format is reliable, valid, and acceptable. Recommendations from the commissioned external review are based primarily on expert opinion, with a limited evidence base comprising data from a small sample acquired during a single examination sitting and without peer review. Unlike the CR2B examination, there is little evidence regarding assessment of the CR1 and CR2 examinations. Both the CR1 and CR2 examinations are currently in the process of undergoing major changes to their formats. Blueprinting items to the curriculum might improve acceptability. Other changes may improve transparency and reliability of these assessments. Our analysis and many aspects of the external review may provide pointers regarding how the upcoming data produced by the “automated” FRCR examinations can be further analysed to provide a more robust evaluation.

## Highlights

• The CR2B examination in its pre-existing format is reliable, valid and acceptable.
• Recommendations from a RCR-commissioned external review are based primarily on expert opinion, with limited evidence base.
• Unlike the CR2B, there is little evidence regarding assessment of the CR1 and CR2.
• Blueprinting items to the curriculum might improve acceptability.
• Future data produced by the “automated” FRCR examinations can be further analysed to provide a more robust evaluation.

## Introduction

The Fellowship of the Royal College of Radiologists (FRCR) examination is the professional qualification that is essential for career progression in clinical radiology within the UK. It is also important for career progression in many countries internationally. The examination currently comprises three parts: Clinical Radiology Part 1 (CR1), Part 2A (CR2A) and Part 2B (CR2B), and has undergone peer-reviewed analyses to determine whether the examination is fit for purpose.^1^, ^2^, ^3^ In 2013, the Royal College of Radiologists (RCR) commissioned an external independent review of all FRCR examinations, which suggested a number of areas for improvement.^4^ Following the release of this report, the RCR made available a commentary section with the description of how the individual recommendations were being addressed.^4^ On a background of the evaluations, the examination has evolved and the format of some of the FRCR examinations has changed. In this systematic review, we appraise and summarise the available data and demonstrate the latest evidence relating to the validity, reliability, and acceptability of the FRCR examination.

## Material and methods

The PubMed and MEDLINE databases were searched for articles published before January 2018 using the major topic MeSH terms ‘‘Educational Measurement” AND “Radiology”. In addition, we examined all available electronic documents from the Royal College of Radiologists. We extracted all relevant statements on the Fellowship of the Royal College of Radiologists and further explored references to capture literature not listed using the MeSH search.

## Results

### FRCR examination structure

The FRCR CR1 examination comprises anatomy and physics papers, both of which must be passed independently. The anatomy paper is composed of 100 multiple choice questions (MCQs), which are based on identifying arrowed anatomical structures on images from a range of imaging techniques, in a range of planes, using a computer work-station. This examination was hand-marked by two examiners until 2017 and is now computer based with the trainees inserting the answer electronically under the “automation” project (described below). The physics paper consists of 40 true/false questions each with a statement and corresponding five-stemmed question. It is answered with a pencil and is also computer-marked. It covers the principles of ultrasound, plain film, computed tomography, magnetic resonance imaging, nuclear medicine as well as radiation safety and protection incorporating Ionising Radiation (Medical Exposure) Regulations legislation.^5^

Success in the CR1 examination allows progression to the CR2A examination, a six-part modular assessment examination linked to the major components of the radiology curriculum. Each module is examined with a separate single best answer (SBA) paper following a change in 2009 from a true/false format.^6^ Candidates can choose a number of modules to take at a single sitting. In the last year, the format of the CR2A examination has changed to a single SBA examination with two papers sat on the same day, each consisting of a 3-hour examination of 120 questions. During an introductory period, both formats run concurrently to allow those on the older format to complete the remaining modules.

The last part of the final CR2B examination is composed of three elements: a rapid reporting session, a long-cases reporting session, and a two-part *viva voce* structured assessment. The rapid reporting session lasts for 35 minutes with the candidate being asked to review 30 radiographs. The cases are chosen to replicate those typically encountered during the day-to-day reporting of the emergency department and general practice examinations. The long-cases reporting session lasts for 60 minutes with the candidate writing a report for six individual cases following a standard format. Each case may involve radiography, computed tomography, fluoroscopy, ultrasound, nuclear medicine, or magnetic resonance imaging. These components have also recently changed under the “automation” project and, instead of using a paper answer sheet, are now fully electronic. During the *viva voce* examination, four examiners test the candidates’ ability to interrogate and interpret a range of radiological studies. The candidate is asked searching questions with the amount of discussion at the discretion of the examiner. The cases may vary with each candidate and there are no set minimum numbers of cases to be reviewed.

### FRCR CR1 examination

The commissioned external review of the FRCR examinations was conducted by a consortium of four reviewers with national and international expertise in the field of assessment in medical education.^4^ None of the reviewers were radiologists. The general conclusions were based on statistical analysis of the Spring 2014 CR1 and CR2 examinations, whilst the CR2B examination was observed by some of the reviewers in the Autumn 2014 examination. Although there were meetings with staff, examiners, and trainees, no mention of their input was detailed in the report. A number of suggestions was made to improve the CR examinations, many of which have a recurring theme throughout their review. We appraise these suggestions and incorporate other evidence, first describing CR1, then CR2, and then the CR2B examination.

The RCR was advised that the development of an overarching statement of purpose for the CR1 examination would be desirable, specifically to describe the purpose of the examination and the application of results. It was recommended that the format of the physics component of the CR1 should change from a true/false to SBA format in line with other examinations, such as the FRCR (Clinical Oncology), to improve reliability and assessment of knowledge. In the past years, the true/false MCQs have declined in number because of the difficulty in creating high-quality questions and because of previously published psychometric evidence relating to gender-related variance and guessing.^4^, ^7^ In addition, areas of apparent suboptimal practice, such as the hand-marking of the anatomy examination, were criticised as a potential source for error, despite being double-marked by two experienced college examiners and despite having access to a third examiner to adjudicate (the senior examiner). No evidence was given to subscribe the risks associated to hand-marking, but these have nonetheless been overcome with the new “automation” project by generating an online response. The automated marking system was appropriately trialled and the candidates are able to practise before sitting the examination. Other suggestions for good practice proposed included the introduction of feedback for both successful and unsuccessful candidates. Since Autumn 2015, both passing and failing candidates were given their score and the pass mark whereas previously they were given to failing candidates only. A theme common to all examination components was a proposal to align examination question topics to the key components of the curriculum, termed “blueprinting”, to improve the defensibility of the examination. It was also noted that the CR1 could be analysed more effectively if the SBA format was implemented rather than true/false questions in the physics examination.

### Final FRCR CR2A examination

The commissioned review used Cronbach α as an index to assess the reliability of the CR2A examination and demonstrated an α value of 0.71–0.78.^4^ Although it was considered by the authors to be just about acceptable, these results were considered low for other well-designed SBA assessments, considering the recommended α >0.8 for selection tests in medicine. These results, however, were obtained on a single sitting; therefore, values may be influenced by (1) The small sample size and (2) the lack of candidate performance variation across several iterations of the examination, each containing differing content.

In line with feedback related to other parts of the FRCR, the commissioned review recommended developing a clear statement of purpose and appropriate alignment of questions to key learning outcomes; in response, statements of purpose have been crafted recently by the RCR. It was noted that anchor questions, which are questions used in a previous examination, are not used to compare the candidate's performance to a previous standard. It was argued that the creation of a master blueprint (examination question topics aligned to the key components of the curriculum) would allow comparison across all CR2A papers and ensure greater consistency between examinations. In addition, as with the CR1 examination, improved mechanisms to deliver feedback were suggested and from 2015 both failing and passing candidates were given feedback regarding the score and pass mark. Other changes to overhaul the CR2A examination were recommended and included instigating a change from the six module format to a single integrated assessment covering the core syllabus. The decision to revert from the CR2A six-module structure to a single examination composed of two papers covering the entire syllabus, was announced in December 2015 and approved by the General Medical Council in the Spring of 2016. The motivation for this change was due to the perception that trainees were focused on studying for examinations for much of their training, to the detriment of clinical training.^8^ In parallel, to reduce the impact on training, recent RCR guidelines specify that the earliest the examination may be taken is in the third year of clinical radiology specialist training.

### Final FRCR CR2B examination

The CR2B examination is a multifaceted examination encompassing rapid reporting, long-case reporting as well as a two-part *viva voce* component and has evolved over the last two decades in response to ongoing review. Previous studies performed between 2011–2014^1^, ^2^, ^3^ have assessed the CR2B component in considerable depth given its complex multi-component nature and its importance as the final hurdle prior to the FRCR award, which allows career progression to consultant status when training has been completed. The key constructs of a high-stakes assessment have been shown to be validity, reliability, and acceptability, which should ideally be used as a means of scrutinising an examination.^1^, ^9^

Yeung *et al.* assessed the acceptability of the CR2B examination with 258 recruited participants who had been candidates in the preceding 44 months.^1^ The participants generally regarded the examination to be fair, acceptable, and valid. Eighty percent agreed that the oral examination was a comprehensive test and demonstrated good construct validity and 63% agreed that the long cases had good construct validity. The examination was shown to have no gender bias or difference in pass rate between those candidates who speak English as a first language and those who are non-native speakers; however, this study did demonstrate a perceived lack of fairness in the long-case component with candidates reporting a lack of time to complete the questions, as well as anxiety related to the *viva voce* component of the examination (albeit there was no difference in pass rate between groups who felt they experienced performance-reducing anxiety and those who did not). Nevertheless, the candidates surveyed had little desire to change the examination format to, for example, an objective structured clinical examination (OSCE)-based format (only 12% favoured that option). Furthermore, 88% of candidates who were assessed considered the present CR2B *viva voce* component representative of day-to-day clinical practice. Content validity, such as that assessed here, can be difficult to incorporate into modern educational theory and its importance may be overlooked. Content validity of the *viva voce* did not appear to be examined by those assessing the commissioned review of the FRCR.

A follow-up study examined 2,235 paired scores from examiners during the *viva voce* component of the CR2B examination to assess reliability.^2^ Statistical analysis using boot-strapping techniques demonstrated a mean difference in paired scores of 0.67 (95% confidence interval [CI]: 0.65–0.70; the maximum score for *viva voce* is 8) with reliability coefficients of 0.27 (weighted Kappa) and 0.44 (intraclass correlation coefficient). This represented fair-to-moderate interobserver variability and reliability of the CR2B examination, which is comparable to other similar postgraduate oral examinations, including the oral components of the Royal College of Physicians (RCP) Membership of the Royal College of Physicians (MRCP) Practical Assessment of Clinical Examination Skills (PACES), Royal College of General Practitioners (RCGP) membership examination (MRCGP),^10^ American Board of Anesthesiology (ABA), and the Royal College of Physicians and Surgeons of Canada (RCPSC) board certifying examinations.^11^ The authors did make suggestions to improve intra-observer and interobserver variability, which included increasing examination duration and making candidates view a set minimum number of cases; however, these changes would likely increase the examination duration and would need to be balanced against examiner and candidate acceptability, validity, and increased cost.

Hawtin *et al.* analysed 2,238 attempts at CRB2 and examined the factors associated with success.^3^ This study used logistic regression analysis to demonstrate no gender or ethnicity bias between candidates from the UK. This finding is an important endorsement for the examination, particularly as gender bias had been shown in other examinations such as MRCP and Fellowship of the Royal College of Anaesthetists^12^, ^13^ and ethnicity bias in other examinations such as MRCP and MRCGP.^13^, ^14^ The study also showed that candidates who underwent UK radiology training obtain higher CR2B marks than candidates who trained abroad at first sitting and were significantly more likely to pass the CR2B at first, second and third attempts. This has been shown to be a common finding in many postgraduate examination analyses and is seen in outcomes related to the MRCP and MRCGP examinations and is thought to relate to differences in international health and education systems.

The commissioned review analysed limited data from only the Spring 2014 sitting with four rapid reporting sets, four long-case sets, and one cohort undertaking the oral component (which gives a mean score for each oral examination). Caveats to interpreting this commissioned review are that the numbers analysed were small, only included first attempt candidates and there was no peer review unlike the CR2B publications described above. Furthermore, it is possible that the participants from the Spring 2014 CR2B may have different characteristics to those sitting the examination in the Autumn^3^ (for example, those candidates who performed better in earlier examination components might be more likely to pass the CR2B examination at the first attempt in an Autumn sitting than those sitting the examination in Spring having re-sat other earlier components). It is also possible that the Autumn 2014 examination may have different characteristics to other CR2B examinations. In summary, therefore, analysis of CR2B data from a single sitting may have missed candidate performance variation across several iterations of the examination, each potentially containing differing content.

The authors of the commissioned review concluded that the CR2B examination was a fair and robust test for the following reasons. The CR2B examination demonstrated content validity in the rapid reporting and reporting sections and was, therefore, felt to be authentic with regards to day-to-day practice. The examination was delivered smoothly and efficiently with good briefing and support for candidates and examiners increasing the acceptability of the examination. Training for examiners and guidance was judged as excellent and information technology risks were considered, minimised, or avoided with considerable preparation and testing. Feedback to candidates after the CR2B examination was shown to be thoughtful and detailed, but only after two attempts at the examination, and hence, it was suggested that this feedback should be given to trainees after the first fail.

The commissioned review noted other aspects of the CR2B examination that were felt could be improved. The CR2B examination should move to a more structured format in-line with the CR1, CR2A, and FRCR (Clinical Oncology) examinations, in particular, the *viva voce* component be replaced with a station-based objective structured clinical examination (OSCE); however, the statistical analysis of the *viva voce* component was limited. For example, the assessment of adjusted (closed) mean and standard deviation scores from all four examiners for each candidate contained no paired examiner scores, unlike the 2,235 paired scores analysed in Yeung *et al.*^2^

Educational theory has evolved to follow certain principles and OSCE-style examinations are the *de rigueur* assessment technique at present^15^, ^16^ by allowing simpler audit analysis, providing uniform scenarios for the candidates, permitting tailoring to the level of skills being assessed and enabling quantification of candidate and assessor performance. Conversion of the *viva voce* to an OSCE-style format might reduce the difficulty of the CR2B examination and reduce the "real-life" simulation of clinical practice. Given that the CR2B examination is designed to simulate clinical practice and demonstrates content validity, there is a risk of making the examination less valid. After all, data from Yeung *et al.* found that 89% of candidates reported that the *viva voce* component of the CR2B examination made them better at their clinical job.^1^ Furthermore, only 12% of candidates thought the examination should move to an OSCE format; therefore, there is a risk of making the CR2B less acceptable too. It is also noteworthy that the *viva voce* component of the current CR2B examination has shown that 85.1% of the scores of paired examiners were within one mark of each other, demonstrating the significant inter-examiner reliability of the examination.^2^

Currently CR2B examiners use prepared cases of their own for the *viva voce* component to cover all pathologies, techniques, and modules with prepared answer sheets. These are all vetted by the Chair of the examination board and together with the two co-examiners, sets are chosen to complement each co-examiner. The authors of the commissioned review thought that the current CR2B model has a design format and construct that allows major examiner-related variance in relation to the examiners' own material and subsequent case selection. They, therefore, suggested that to improve consistency, transparency, and fairness, the cases for the *viva voce* component of the CR2B examination should be written centrally in a similar model to the Clinical Oncology 2B examination. The commissioned review also recommended that the cases for the CR2B examination components should be blueprinted (examination question topics aligned to the key components of the curriculum), again because of the perception of the current excessive reliance on the examiners skills and their ability to select their own material. In response to these recommendations the RCR has agreed to include a proportion of centrally standardised cases for each examination pair during the *viva voce* component^4^ in order to reduce variance between stations; therefore, each candidate would see at least some standard cases at the start of each *viva voce*.

One of the main strengths of the *viva voce* component of the CR2B examination is its flexibility and adaptability depending on the candidate's responses mimicking the daily interactions of radiologists with clinician. Therefore, the standardisation may reduce the validity of the *viva voce* component of the CR2B examination. In summary, although the move towards a more standardised assessment for the CR2B examination will facilitate the generation of more accurate psychometric data to analyse the assessment process, the authors present no evidence to demonstrate that this will provide a more accurate assessment of clinical radiological acumen, or indeed a more proficient radiologist.

The commissioned review considers the use of a 4–8 assessment scale confusing with no recognised clarity of purpose behind the scaling, and suggests adding an overall grade based in letter/symbol rather than a numeric format. The RCR commentary response was to consider domain-based scoring (awards marks in domains of practice, such as description of imaging type or description of abnormality and allows more flexibility), in order to obtain more data and consequently improve reliability and analysis.

The current CR2B marking system uses a criterion-referenced method whereby a pass mark is set rather than a norm-referenced method whereby a set proportion of candidates pass on each sitting. Criterion-referenced assessments are intended to measure a candidate's performance against a fixed predetermined set of standards. The CR1 and CR2A examinations, but not the CR2B examination, use a criterion-referenced modified Angoff approach, which relies on expert judgement to assess the difficulty of each item.^17^ Norm referencing compares fellow candidate's performance and removes the subjective judgement element of the expert examiner, but does not take into account the difficulty rating of individual items.

The CR2B criterion-referenced standard was also criticised as it uses an arbitrarily fixed passing score of 75%, deemed questionable given the amount of potential variance between test material, examiner pairing, and candidate factors. As previously stated, one of the commissioned review suggestions is to change the oral examination to a more structured format closer to the OSCE model used in Clinical Oncology in which case either the Borderline method (a person-centred method) or the Angoff method (an item-centred method) could be considered as tools for criterion-referenced standard setting. Rather than the items that differentiate competent candidates, person-centred studies evaluate the examinees themselves, which can be more challenging as examinees are not a static group as a list of items is. In addition, note that the Angoff method is considered suitable for both large and small cohorts, unlike the Borderline method, which is only adequate for a total candidate number >50. An amendment to the criterion referencing may improve the defensibility of the examination.

There is no evidence that the commissioned review explored the acceptability or validity of the current examination, nor is there evidence that the reviewers discussed their suggested changes regarding the examination curriculum with participants of the clinical radiology examination. Ideally, a comprehensive review of the clinical radiology should seek the opinion of all stakeholders involved.

Statistical analysis of four rapid reporting sets (234 candidates) from the CR2B examination from Spring 2014 by the commissioned review demonstrated weak-to-low reliability coefficients (Cronbach α scores 0.27–0.65) with high mean scores (83.9–91.1%). The reliability coefficients are low, which might reflect several subtle radiographic abnormalities within the set of 30 radiographs. It is possible that this might represent real clinical practice where interpretation can be challenging, but is nonetheless important; however, given the paucity of data, it is reasonable to assume that the Cronbach α results were artificially low secondary to a small sample size.

Rudimentary interpretation of the high mean scores might have led the reviewers to suggest that the rapid reporting component is too easy given the importance of this high-stakes assessment; however, high mean scores are expected as the referenced pass mark is high (minimum 27/30; 90%). The marking scheme relates to content validity, with high accuracy expectations in real clinical practice (i.e., safety) aligned with reporting efficiency (hence “rapid” reporting). Moreover, contrary to the commissioned review findings, a large analysis of 2,238 CR2B examination attempts over 5 years demonstrated that pass rates have declined over this time period with pass rates of 65.7% in 2006 falling to 54.5% in 2010,^3^ making it plausible that the overall difficulty of the CR2B examination has not diminished.

A sub-analysis was performed by the commissioned review to assess the difference in mean scores between the four rapid reporting sets in a small number of cases. Significant differences in mean scores were found between sets for which there are a number of potential causes, including differences in cohort ability or set difficulty. Unfortunately, the commissioned review did not compare these data with other CR2B components (long-cases and rapid reporting), which might have helped to determine whether there was a difference in cohort ability. The review commented on the potential for variance in rapid reporting set difficulty due to the lack of standard setting; however, it is unclear whether the suggested changes to standard setting would achieve the desired homogeneity in rapid reporting cohorts (and conceivably may have a negative effect on the CR2B examination). Nonetheless, the RCR assessed the feasibility of introducing modified standard setting for the long-cases and rapid reporting components, which has now become more feasible following the introduction of the “automation” of the FRCR for the Spring 2018 sitting.

The four long-cases reporting sets from 233 candidates were also analysed by the commissioned review and demonstrated lower mean scores with smaller standard deviations than the rapid reporting component (mean scores 71.4–75.5, SD 5.2–6.4; compared to 83.9–91.1, SD 6.2–8.6), but like the rapid-reporting component had low mean item discrimination (the ability of an item to differentiate among candidates on the basis of how well they know the material being tested) and low Cronbach α scores (0.44–0.56; compared to 0.27–0.65). Again, the low Cronbach α scores might be artificially low due to the small sample size. A greater number of items might improve the reliability should a low Cronbach α score persist despite a bigger sample size. The mean score difference between the long-cases and rapid reporting components was postulated to suggest a lack of consistency between the two examination components, i.e., the rapid reporting was perceived to be easier than the long-cases component; however, each component contains different subject matter. The long cases are a test of observation, interpretation, and clinical knowledge of complex multi-technique imaging and require a different marking scheme compared to rapid reporting.

Collective analysis of all components (including rapid reporting, long-case reporting, and the *viva voce* examinations) demonstrated a mean score of 73%, with a standard deviation of 6.52% and Cronbach α score of 0.57. Although the commissioned review assessment of reliability of the whole 2B examination is useful, the low score is likely to be technical because only four items were used for the reliability calculation.

## Discussion

The commissioned external review adds to the current paucity of literature evaluating all parts of the FRCR examination. The recommendations therein are based primarily on expert opinion, with limited evidence base comprising data from a small sample acquired during a single examination sitting and without peer review. The recommendations have not been “tried and tested” in the specialty of clinical radiology, nor has input from the key stakeholders been demonstrated. The peer-reviewed literature describing the analysis of large CR2B datasets has also not been considered in the review. Although there are many sensible recommendations within the review based on the established assessment literature, it is noteworthy that the CR2B examination in its pre-existing format has already been shown to be reliable, valid, and acceptable. Importantly for a postgraduate examination, the CR2B examination does not demonstrate gender or ethnic bias as has been shown in the MRCP, MRCGP, and FRCA examinations. We suggest that the recommendations of the commissioned review should be interpreted with caution, and a comprehensive evaluation process should be incorporated before any planned future change to the structure or format of the CR2B.

Unlike the CR2B examination, there is little evidence regarding assessment of the CR1 and CR2 examinations. Both CR1 and CR2 examinations are currently in the process of undergoing major changes to their formats. Indeed, the new CR2A examination format (single examination) has addressed concerns shared by the RCR and the commissioned review. Blueprinting items to the curriculum is sensible and might improve acceptability. Other changes may improve transparency and reliability of these assessments. With the new “automation” project converting all the written components of the FRCR into an electronic format, new data will be available and will lead to documentary and governance enhancements.

In summary, it does not appear that there is sufficient evidence to prove that the pre-existing FRCR (in particular the CR2B) examinations are not already valid, acceptable, and reliable. Our analysis and many aspects of the external review may provide pointers regarding how the upcoming data produced by the “automated” FRCR examinations can be further analysed to provide a more robust evaluation.
